# Supplementary material for: Effects of abscisic acid on growth and selenium uptake in medicinal plant Perilla frutescens
Source: PLoS One. 2022 Oct 7;17(10):e0275813. doi: 10.1371/journal.pone.0275813 (PMC9543941; doi:10.1371/journal.pone.0275813)
Supplement: Attachment — Submitted filename: review.doc [file pone.0275813.s002.doc]

Reviewers' comments:

5. Review Comments to the Author

Please use the space provided to explain your answers to the questions above. You may also include additional comments for the author, including concerns about dual publication, research ethics, or publication ethics. (Please upload your review as an attachment if it exceeds 20,000 characters)

Reviewer #1: Well-designed and carried out research work has yielded beautiful and even useful and timely results for practice. Its main goals (see 2 p./26-27.) were achieved by the work. In addition, it has been demonstrated that „ABA may improve resistance of P. frutescens to soil Se stress by increasing antioxidant enzyme activity and reducing membrane lipid peroxidation.”
Thank you for your reviewing.

MS uses a relatively large number of abbreviations (ABA, SOD, POD, CAT, MDA, TF, TAF, BCF). Each of them receives an explanation at the first time of their application, but in different parts of the MS. Therefore, it may be suggested to create an Abbreviations chapter after the Keywords chapter, before the Introduction chapter. The inscriptions in Table 3 and Table 7 do not include explanations of abbreviations.
We have added the Abbreviations section, and the abbreviations are also added in Table 3 and Table 7.

Ad 2 p. 27:
Please clarify the following thought/sentence: "to provide a basis for P. frutescens production. (production development ?, ?).
It should be production development. We have revised.

Letter and character errors:
ad 2nd p./ 24 (Corrected by Liao, 2018)
ad 2nd p./ 24 (Correct: Thlaspi arvense)
ad 12 p./ 5.
ad 13 p./ 16.
give. p. 13/ 13.
ad 14 p./ 7.
We have revised.

The Latin phrase Per capita (ad 1 p./29), as a text in a foreign language, is recommended to be highlighted, written in italics.
We have revised.

Literature:
It is recommended to distinguish between the two Li et al., 2019 citations (for example: a, with b notations)

We have revised.

They are included in the literature, but there is no citation in the text for the following two articles: Liu et al., 2009; Yi et al., 2020

We have deleted them.

Guignardi et Schiavon (2017): place of publication? (Cham?)

New York, USA. We have added.

Latin name to highlight: p. 12 / 19.; 13. p./ 2 (chinensis)

We have revised.

Shao et al. citation does not seem complete.

It may be the wrong citation. We have replaced by “Cui L, Zhao J, Chen J, Zhang W, Gao Y, Li Ba, Li YF (2018) Translocation and transformation of selenium in hyperaccumulator plant *Cardamine enshiensis* from Enshi, Hubei, China. Plant and Soil 425: 577-588. ”

Twice-featured citation: Wang et al., 2016.

We have deleted onr.

Zhang et al. (2021) citation correct? In internet searches, the reviewer could not find it.

It may be the wrong citation. We have replaced by “Sah SK, Reddy KR, Li J (2016) Abscisic acid and abiotic stress tolerance in crop plants. Frontiers in Plant Science 7:571.”

Reviewer #2: The tasks set correspond to the objectives of the study. The presented data allow us to assess the reliability of the work. The study was conducted qualitatively, but I think that such a result is expected. Concentrations of ABA can enhance the adaptation of plants to drought by inhibiting growth processes. Therefore, the authors did not receive a significant increase in growth characteristics. It would be interesting to consider the mechanism affecting the absorption of selenium. The research is interesting, but its significance is small. The work can be published because it may be of interest to young scientists.

Thank you for your reviewing.
